# Methanol extract of Iraqi Kurdistan Region *Daphne mucronata* as a potent source of antioxidant, antimicrobial, and anticancer agents for the synthesis of novel and bioactive polyvinylpyrrolidone nanofibers

**DOI:** 10.3389/fchem.2023.1287870

**Published:** 2023-10-25

**Authors:** Khursheed Muzammil, Mazin Hadi Kzar, Faraj Mohammed, Zahraa Ibrahim Mohammed, Sarah A. Hamood, Talib Kh. Hussein, Saheb Jubeir Hanoon, Maytham T. Qasim, Ahmed Hussien Alawadi, Ali Alsalamy

**Affiliations:** ^1^ Department of Public Health, College of Applied Medical Sciences, Khamis Mushait Campus, King Khalid University, Abha, Saudi Arabia; ^2^ College of Physical Education and Sport Sciences, Al-Mustaqbal University, Hillah, Iraq; ^3^ Department of Medical Laboratories, Al-Manara College For Medical Sciences, Maysan, Iraq; ^4^ Department of Radiology and Sonar Techniques, Al-Noor University College, Nineveh, Iraq; ^5^ Department of Medical Engineering, Al-Esraa University College, Baghdad, Iraq; ^6^ Department of Medical Laboratories, Al-Hadi University College, Baghdad, Iraq; ^7^ Department of Medical Laboratories, College of Health and Medical Technology, Sawa University, Almuthana, Iraq; ^8^ Department of Anesthesia, College of Health and Medical Technololgy, Al-Ayen University, Thi-Qar, Iraq; ^9^ College of Technical Engineering, The Islamic University, Najaf, Iraq; ^10^ College of Technical Engineering, The Islamic University of Al Diwaniyah, Al Diwaniyah, Iraq; ^11^ College of Technical Engineering, The Islamic University of Babylon, Babylon, Iraq; ^12^ College of Technical Engineering, Imam Ja’afar Al-Sadiq University, Al-Muthanna, Iraq

**Keywords:** methanolic extract, Iraqi Kurdistan Region *Daphne mucronata*, polyvinylpyrrolidone nanofibers, antioxidant activity, antimicrobial activity, anticancer activity

## Abstract

In this study, aqueous, ethanol, methanol, and hexane extracts from Iraqi Kurdistan Region Daphne mucronata were prepared due to the numerous applications and development of nanofibers in biological and medical fields, including food packaging, enzyme stabilization, and wound dressing. In the initial evaluation of the extracts, the antioxidant properties against DPPH, antimicrobial properties against 3-gram-positive bacterial species, 3-gram negative bacterial species, 3-common bacterial species between aquatic and human, and 3-fungal species, and anticancer properties against breast cancer cells were performed. The results proved that the methanol extract has the highest antimicrobial, antifungal, antioxidant, and anticancer properties. After identifying the compounds of prepared methanol extract using GC/MS, polyvinylpyrrolidone nanofibers containing methanol extract of *Daphne mucronata* were prepared. The structure and characteristics of prepared nanofibers were confirmed and determined using FTIR, TGA, BET, SEM, flexural strength, compressive strength, and hydrophilicity. Synthesized polyvinylpyrrolidone nanofibers containing methanol extract of *D. mucronata* were subjected to antimicrobial properties on the strains studied in methanol extract of *D. mucronata*. The antimicrobial properties of synthesized polyvinylpyrrolidone nanofibers containing methanol extract of *D. mucronata* were compared. The results showed that synthesized polyvinylpyrrolidone nanofibers containing methanol extract of *D. mucronata* have the potential to introduction bioactive natural synthesis nanoparticles.

## 1 Introduction

Today, using natural compounds and extracts has found a special place in treating diseases. By examining the previous texts, we realize that the extracts of different plants have many and varied biological properties. The most crucial property of plant extracts is their antioxidant activity. This activity can be attributed to the presence of flavonoids in most plants. Oxidants are the cause of cancer, and in a way, anti-cancer properties can be attributed to plants with high antioxidant properties. However, there have been reports of the anticancer properties of extracts that have been directly subjected to anticancer tests. For example, the anticancer properties of *Adenosma bracteosum* extract were reported in 2020. In addition, other biological properties such as anti-bacterial activity, anti-inflammatory activity, anti-mutagenic activity, immunostimulatory activity, and anti-tumoral activity have been reported from plant extracts (Mostafa et al., 2018; Dirar et al., 2019; Manandhar et al., 2019; Tanase et al., 2019; Nguyen et al., 2020; Dias et al., 2021).

Different species of the genus *Daphne* are distributed in many countries, especially in South America, Asia, and Europe and around the Mediterranean Sea. *Daphne giraldii* and *Daphne mucronata* are two important species of *Daphne* with unique biological properties. Anticancer properties against liver cancer cells, antimalarial, hypnotic and sedative, anti-nociceptive, anti-inflammatory, and immunomodulating properties of *D. giraldii* have been reported (Sun et al., 2017; Han et al., 2020). Biological properties such as inhibition of STAT3 and Smad3/4 cancer, nephrotoxicity and hepatotoxicity, antibacterial activity, and antioxidant activity have been reported for *D. mucronata* from different regions (Shah et al., 2018; Lutfullah et al., 2019; Ghanadian et al., 2020; Nazir et al., 2021).

The use of environmentally friendly plant and polymer extracts and the preparation of new nanoparticles in the form of nanofibers has been the focus of scientists in recent years. Numerous nanofibers containing plant extracts or other nano compounds can be synthesized using electrospinning technology. The performance and properties of nanofiber extracts can be improved by using this method. Nanofibers increase the activity of the extracts for two crucial reasons, including the nanosizing of the compounds and the increase in the specific active surface area that leads to contact with the desired agents. Natural nanofibers, mainly composed of biodegradable and environmentally friendly polymer compounds such as polyvinylalcohol and polyvinylpyrrolidone, have many biological properties and industrial applications. Applications of nanofibers in biological and medical fields include food packaging, enzyme stabilization, and wound dressing (Park et al., 2019; Kurakula and Rao, 2020; Mehrali et al., 2020; Osanloo et al., 2020; Yadav et al., 2020; Dodero et al., 2021; Ghasemian Lemraski et al., 2021; Li et al., 2022; Wu et al., 2022; Kişla et al., 2023; Wu et al., 2023).

For developing and reporting nanofibers with biological properties and synthesis of new nanofiber compounds, aqueous, ethanol, methanol, and hexane extracts of *D. mucronata* from the Iraqi Kurdistan Region were prepared. After identifying the active compounds in the extract, biological evaluations such as its antibacterial, antioxidant, and anticancer properties were investigated and compared. Nanofibers that contained Iraqi Kurdistan Region Daphne mucronata and polyvinylpyrrolidone were synthesized using the electrospinning method. After characterizing and confirming the structure, the antimicrobial properties of the synthesized nanofibers were also evaluated, and the results obtained were compared with the extract.

## 2 Materials and methods

### 2.1 Materials

Agilent GC/MS-3223 with HP5 capillary column 30 m long was used to identify the compounds of the extract. In microbiology and antioxidant tests, the Unico S2150 spectrophotometer was used to prepare the concentration of strains and measure absorbance, respectively. BIOTEK ELX800TS Elisa Reader was used in the anticancer trials of the extracts.

### 2.2 Preparation of *Daphne mucronata* extracts

*Daphne mucronata* was collected from the mountains of the Iraqi Kurdistan Region. After that, the leaves were washed with distilled water and dried for a week at room temperature without sunlight. Then, it was made into powder by an electric mill. To prepare an aqueous, ethanol, methanol, and hexane *D. mucronata* extracts, 1 to 10 wt% powder to solvent was stirred for 48 h, at ambient temperature and in the dark. Finally, condensation took place at a temperature of 37°C (Fazal et al., 2020).

### 2.3 Identification of the compounds in the *Daphne mucronata* extracts

GC/MS technique was used to identify the compounds of the extract. The method used includes a thermal program from 50°C to 280°C with an increase in temperature of 4°C per minute; helium carrier gas with a flow of 1.5 mL per minute; the temperature of the injection chamber and the temperature of the detector is 270°C; and the split ratio was 10:1. The mass spectrum of the compounds was recorded at 70 eV with a mass range of 50–470 amu. Electronic integration was used to obtain information from the surface under the peaks. Identifying the extract’s compounds was achieved by comparing the obtained mass spectra and inhibition indices with those of the standard compounds (Popiel et al., 2014; Rhazi et al., 2022).

### 2.4 Antimicrobial tests

In microbiology tests, the guidelines and standards of the Clinical and Laboratory Standards Institute for measuring Minimum Inhibitory Concentration value, Minimum Bactericidal Concentration value, Minimum Fungicidal Concentration value, and Inhibition Zone Diameter value were used (Etemadi et al., 2016; Ahani et al., 2018; Hosseinzadegan et al., 2020).

### 2.5 Antioxidant tests

Previous studies were used to measure the antioxidant properties of the extracts using the DPPH method at a concentration range of 5–20 g/mL. The equation presented in Section 3.3 was utilized to calculate inhibition (%) (Beyzaei et al., 2018; Moghaddam-manesh et al., 2021).

### 2.6 Anticancer tests

MTT assay protocol or Thiazolyl Blue method was used to evaluate the anticancer activity of the extracts on breast cancer cells (MCF-7). For this purpose, previous reports were used (Akhavan-Sigari et al., 2022; Moghaddam-manesh et al., 2022).

### 2.7 Synthesis of polyvinylpyrrolidone nanofibers containing methanol extract of *Daphne mucronata*

To prepare polyvinylpyrrolidone nanofibers containing methanol extract of *D. mucronata*, the first polymer solution of polyvinylpyrrolidone and methanolic extract of *D. mucronata* was prepared. For this purpose, a polymer solution with a 12% w/w of polyvinylpyrrolidone in acetic acid was prepared based on previous studies. Then 2 mg of the *D. mucronata* extract was added to 10 mg of the prepared polyvinylpyrrolidone polymer. It was stirred for 2 h at room temperature and in the dark for uniformity. Then, electrospinning was done under a voltage of 25 kV, a flow rate of 0.4 mL/h, and a spinning distance of 20 cm (Edikresnha et al., 2019).

## 3 Results and discussion

### 3.1 Identifying the compounds in *Daphne mucronata* extracts

Aqueous, ethanol, methanol, and hexane extracts of *D. mucronata* were made in this study. Antimicrobial and antioxidant tests were performed on the prepared extracts. Section 2.3 and Section 3.3 detail the biological results of the prepared extracts.

In general, it was found that the methanol extract has the most biological activity. The compounds in the methanol extract were obtained using GC/MS and the method presented in Section 2.3. The GC/MS results identified 17 compounds in this extract. The highest percentage of compounds were related to Gallic acid, Catechins, and Hesperidin, respectively.

The structure of the main compounds identified, the amount, and retention time are given in Table 1.

**TABLE 1:** The main compounds identified in the methanol extract of *Daphne mucronata*.

The name of the identified compound	The structure of the identified compound	Retention time (min)	Amount (mg/L)
Gallic acid	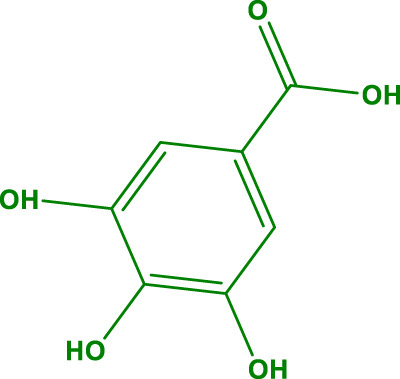	3.4	137
Catechins	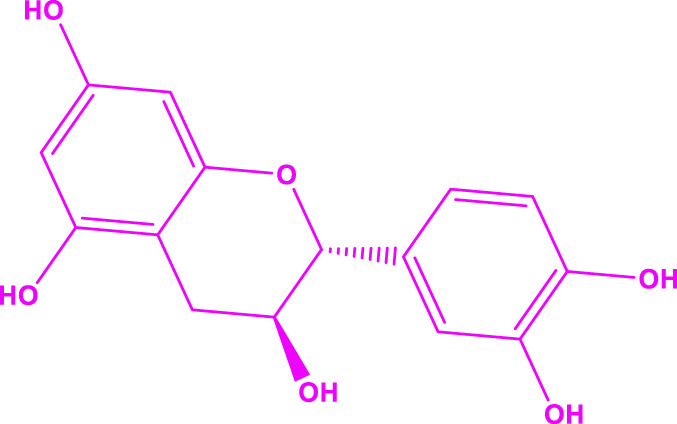	8.2	1,532
Hesperidin	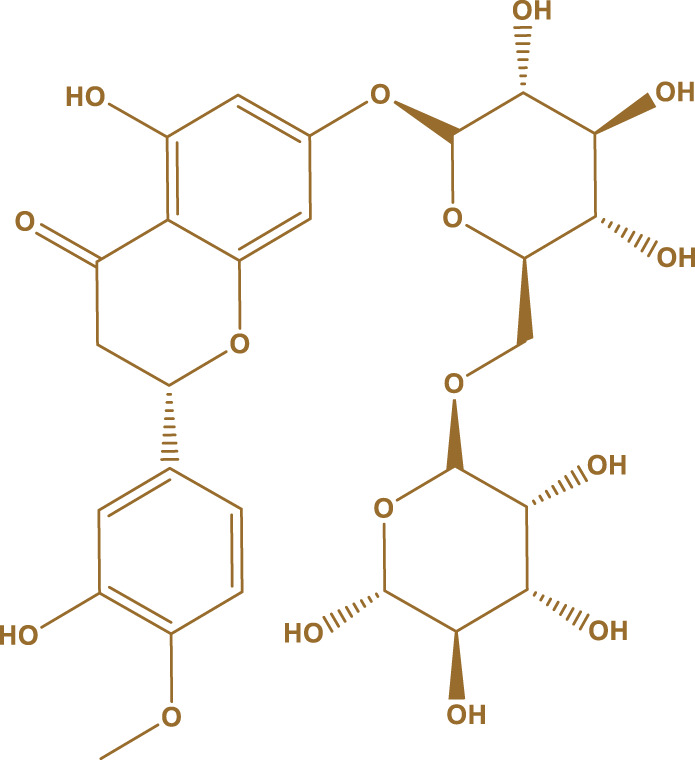	18.7	1,067

### 3.2 Antimicrobial evolution of *Daphne mucronata* extracts

Antimicrobial evolution of aqueous, ethanol, methanol, and hexane *D. mucronata* extracts on 3-gram-positive bacterial species, 3-gram negative bacterial species, 3-common bacterial species between aquatic and human, and 3-fungal species was performed. The results of the tests are given in Table 2. The studied gram positive bacterial, gram negative bacterial, common bacterial between aquatic and human and fungal species are *Bacillus cereus* (ATCC 11778), *Rhodococcus equi* (ATCC 25729), *Staphylococcus aureus* (ATCC 29213); *Klebsiella pneumoniae* (ATCC 13883), *Yersinia* enterocolitica (ATCC 9610), *Acinetobacter baumannii* (ATCC 19606); *Yersinia ruckeri* (ATCC 29473), *Loctococcus garvieae* (ATCC 43921), *Streptococcus iniae* (ATCC 29178); and *Fusarium oxysporum* (ATCC 7601), *Candida albicans* (ATCC 10231), *Aspergillus fumigatus Fresenius* (ATCC 1022), respectively.

**TABLE 2:** Antimicrobial activity of aqueous, ethanol, methanol, and hexane *Daphne mucronata extracts*.

Extracts species			Aqueous extract	Ethanol extract	Methanol extract	Hexane extract
Gram-positive	ATCC 11778	IZD	11.37	15.06	15.27	9.94
		MIC	1,024	128	64	1,024
		MBC	2,048	256	128	2,048
	ATCC 25729	IZD	—	—	—	—
		MIC	—	—	—	—
		MBC	—	—	—	—
	ATCC 29213	IZD	12.73	13.87	16.49	11.01
		MIC	1,024	512	128	1,024
		MBC	2,048	1,024	256	2,048
gram negative	ATCC 13883	IZD	—	11.62	12.04	10.83
		MIC	—	512	64	2,048
		MBC	—	1,024	128	4,096
	ATCC 9610	IZD	11.53	14.88	19.05	—
		MIC	2,048	512	128	—
		MBC	4,096	1,024	256	—
	ATCC 19606	IZD	—	—	—	—
		MIC	—	—	—	—
		MBC	—	—	—	—
aquatic and human	ATCC 29473	IZD	—	—	9.79	—
		MIC	—	—	256	—
		MBC	—	—	512	—
	ATCC 43921	IZD	—	—	14.34	—
		MIC	—	—	256	—
		MBC	—	—	512	—
	ATCC 29178	IZD	12.18	12.75	14.91	11.27
		MIC	1,024	256	128	1,024
		MBC	2048	512	256	2048
fungal	ATCC 7601	IZD	12.92	13.67	15.73	10.37
		MIC	512	256	64	512
		MFC	512	512	128	1,024
	ATCC 10231	IZD	—	—	12.61	—
		MIC	—	—	512	—
		MFC	—	—	1,024	—
	ATCC 1022	IZD	—	9.21	14.32	—
		MIC	—	2048	256	—
		MFC	—	4,096	512	—

MIC, MBC, and MFC, μg/mL; IZD, mm.

Based on the results obtained and presented in Table 2, the methanol extract of *D. mucronata* was most effective on the studied bacterial and fungal species. In this extract, MIC between 64 μg/mL- 256 μg/mL, MBC between 128 μg/mL- 512 μg/mL and IZD between 9 mm and 19 mm on studied bacterial species were observed, and MIC between 64 μg/mL- 512 μg/mL, MFC between 128 μg/mL- 1,024 μg/mL and IZD between 12 mm and 15 mm on the studied fungal species were observed.

The review of the literature proves that Gallic acid and Catechins have antimicrobial properties, including strong antibacterial and antifungal properties (Górniak et al., 2019; Ma et al., 2019; Rajamanickam et al., 2019; Panda and Duarte-Sierra, 2022; Shahryari et al., 2022) the effectiveness on the studied strains can be attributed to the high amount of their in the extract.

### 3.3 Antioxidant evolution of *Daphne mucronata* extracts

The DPPH method was used to specifically investigate the antioxidant properties of aqueous, ethanol, methanol, and hexane *D. mucronata* extracts.

In the studies, ascorbic acid was used as a known natural antioxidant compound to compare the antioxidant properties of the extracts.

The results obtained from antioxidant tests are given in Table 3.

**TABLE 3:** Antioxidant activity of aqueous, ethanol, methanol, and hexane *Daphne mucronata extracts*.

ExtractsConcentrations (μg/mL)	Inhibition (%)				
	Aqueous extract	Ethanol extract	Methanol extract	Hexane extract	Ascorbic acid
5	32.75	45.86	81.66	29.79	89.47
10	39.42	48.13	87.89	32.48	91.15
15	51.99	54.76	92.17	42.05	91.33
20	60.06	73.16	94.06	56.17	92.29
IC_50_	14.72	11.81	4.67	17.18	3.87

In tests, adsorption of DPPH solution and DPPH solution containing extracts were measured, and inhibition (%) and IC_50_ were calculated. To calculate inhibition (%), Eq. 1 was used.

$$\text{Percent inhibition }\left(\%\right)=\left[\left(A\text{ related to DPPH}-A\text{ related to mixture of DDPH and sample}\right)/\left(A\text{ related to DPPH}\right)\right]\times 100$$ (1)

A=Absorption

To calculate IC_50_, the curves of inhibition (%), and the concentration IC_50_ were calculated. IC_50_ for aqueous, ethanol, methanol, and hexane *D. mucronata* extracts were obtained as 14.72 μg/mL, 11.81 μg/mL, 4.67 μg/mL, and 17.18 μg/mL, respectively.

The antioxidant property of methanol extracts of *D. mucronata* was very close to ascorbic acid (3.84 μg/mL). High amounts of Gallic acid polyphenol and Hesperidin bioflavonoid, which are known as compounds with potent antioxidant properties (Alavi Rafiee et al., 2018; Binkowska, 2020; Lee et al., 2020; Zahrani et al., 2020), can cause high antioxidant activity of the extract.

### 3.4 Anticancer evolution of *Daphne mucronata* extracts

**FIGURE 1:**
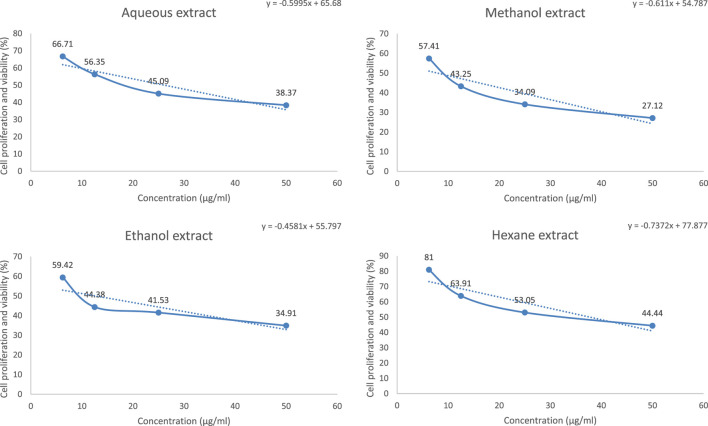
Cell proliferation and viability/concentration of aqueous, ethanol, methanol, and hexane *Daphne mucronata extracts* against anti-breast cancer cells (MCF-7) (*n* = 3).

The anticancer activity of aqueous, ethanol, methanol, and hexane *D. mucronata* extracts against breast cancer cells was measured at 48 h at different concentrations (6.25 μg/mL- 50 μg/mL). Cell proliferation and viability of the extracts in various concentrations are given in the diagram of Figure 1.

Based on the results, it was proved that the lowest cell proliferation and viability are related to the methanol extract.

Using the concentration and cell proliferation and viability graph, the amount of IC_50_ was measured for the extracts, the results of which are given in Table 4.

**TABLE 4:** IC_50_ values of aqueous, ethanol, methanol, and hexane *Daphne mucronata extracts* in anti-breast cancer cells (MCF-7) activity.

Entry	Extracts	Calculated IC_50_ (μg/mL)
1	Aqueous extract	26.15
2	Ethanol extract	12.65
3	Methanol extract	7.83
4	Hexane extract	37.81

The lowest IC5_0_ value was observed for methanol extract at 7.83 μg/mL. According to previous studies, the IC5_0_ of abemaciclib, a breast cancer drug, has been reported using the MTT method of 7.29 μg/mL (Anwer et al., 2022). The IC5_0_ value observed for the methanol extract is very close to the Abemaciclib.

### 3.5 Confirming the structure and determining the characteristics of polyvinylpyrrolidone nanofibers containing methanol extract of *Daphne mucronata*

In general, as the results of Section 3.2 and Section 3.3 showed, the antimicrobial, antioxidant, and anticancer properties of methanol extract of *D. mucronata* were more than other extracts. Therefore, in continuation of our studies on *D. mucronata* extract, polyvinylpyrrolidone nanofibers containing methanol extract *of D. mucronata* were synthesized by electrospinning method.

**FIGURE 2:**
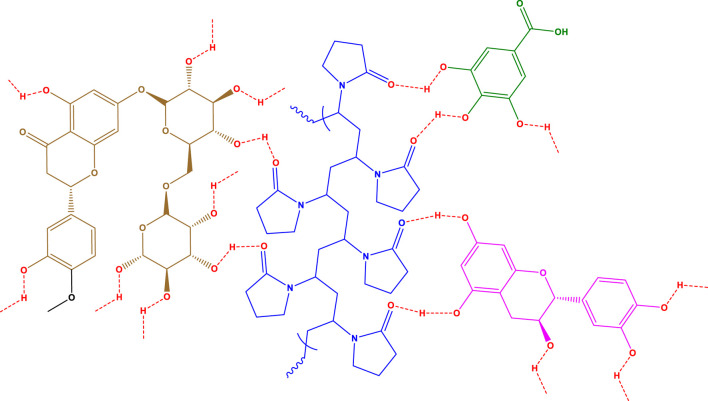
Proposed structure for polyvinylpyrrolidone nanofibers containing methanol extract of *Daphne mucronata*.

The carbonyl oxygen of Polyvinylpyrrolidone can establish hydrogen bonds with alcoholic hydrogens of Gallic acid, Catechins, and Hesperidin, and the structure of Figure 2 can be proposed for the synthesized polyvinylpyrrolidone nanofibers containing methanol extract *of D. mucronata*.

The structure and characteristics of prepared nanofibers were confirmed and determined using FTIR, TGA, BET, SEM, flexural strength, compressive strength, and hydrophilicity.

**FIGURE 3:**
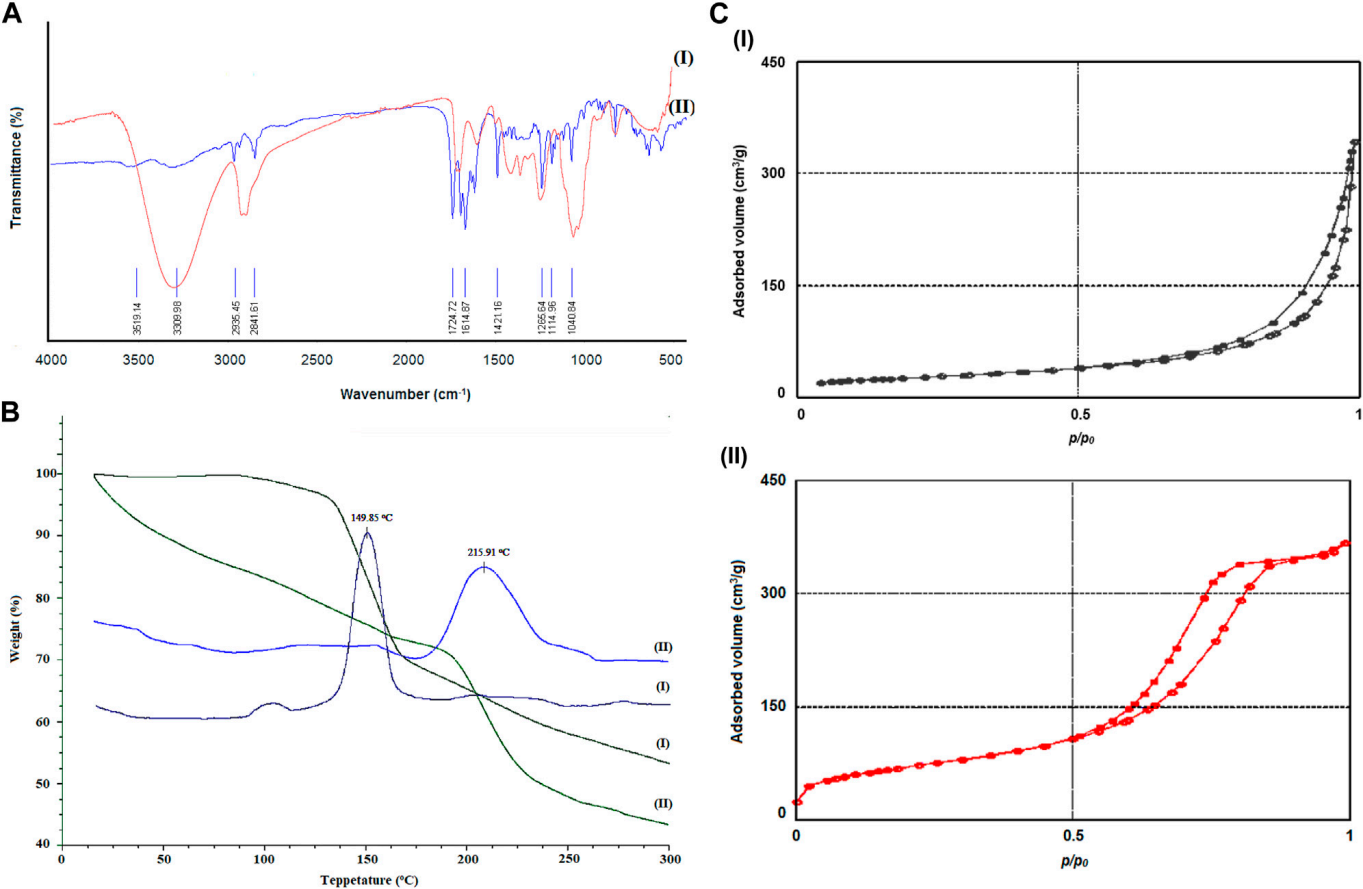
FT-IR **(A)**, TGA/DTG curves **(B)**, and N_2_ adsorption/desorption isotherms **(C)** of polyvinylpyrrolidone nanofibers (I) and polyvinylpyrrolidone nanofibers containing methanol extract of *Daphne mucronata* (II).

FT-IR of polyvinylpyrrolidone nanofibers (I) and polyvinylpyrrolidone nanofibers containing methanol extract of *D. mucronata* (II) dose is shown in Figure 3A. As can be seen in the spectrum of the final product, peaks related to O-H groups in the region of 3,300–3,500 cm^−1^, C-H groups in the area of 2,800–3,000 cm^−1^, and C=O groups in the region of 1,600–1725 cm^−1^ are visible. From the comparison of the two spectra in the mentioned areas, it can be concluded that the proposed product of Figure 1 is formed. For example, in the spectrum of the final product, two broad peaks can be observed in the region of 3,300 cm^−1^ to 3,500 cm^−1^, one of which can be attributed to the alcoholic hydroxyl groups and the second one can be attributed to the carboxylic acid group of Gallic acid. Other spectra differences in the area below 3,000 cm^−1^ and in the region 1,600 cm^−1^ to 1725 cm^−1^, can be attributed to C-H groups related to Catechins and carbonyls related to Hesperidin.

The TGA/DTG Curves, which show the thermal stability of compounds polyvinylpyrrolidone nanofibers (I) and polyvinylpyrrolidone nanofibers containing methanol extract of *D. mucronata* (II), are shown in Figure 3B. As can be seen from the comparison of the curves, polyvinylpyrrolidone nanofibers containing methanol extract of *D. mucronata* have more thermal stability than polyvinylpyrrolidone. From examining of the breaks of 3-B-II, it can be suggested that the weight loss up to the temperature of 150°C is related to methanol and acetic acid molecules trapped in the final composition. Weight loss in areas above 200°C can be attributed to the destruction of organic compounds in synthesized polyvinylpyrrolidone nanofibers containing methanol extract of *D. mucronata*.

The specific surface area of polyvinylpyrrolidone nanofibers (I) and polyvinylpyrrolidone nanofibers containing methanol extract of *D. mucronata* (II) was obtained using the N_2_ adsorption/desorption isotherm presented in Figure 3C. The specific surface area of polyvinylpyrrolidone nanofibers and polyvinylpyrrolidone nanofibers containing methanol extract of *D. mucronata* was obtained as 780 m^2^/g and 1,025 m^2^/g, respectively (Table 5). Nanoparticles’ unique properties, applications, and capabilities are caused by the specific surface area, as we know (Baig et al., 2021; Naseem and Durrani, 2021). In this study, the high specific surface area has led to an increase in the microbiological properties of polyvinylpyrrolidone nanofibers containing the methanol extract of *D. mucronata* compared to the methanol extract of *D. mucronata*, which is discussed in detail in Section 3.6.

**TABLE 5:** BET, BJH volume pore and Mean pore diameter of Polyvinylpyrrolidone nanofibers and Polyvinylpyrrolidone nanofibers containing methanol extract of *Daphne mucronata*.

Sample	BET (m^3^/g)	BJH volume pore (cm^3^/g)	Mean pore diameter (nm)
Polyvinylpyrrolidone nanofibers	780	0.31	1.17
Polyvinylpyrrolidone nanofibers containing methanol extract of *Daphne mucronata*	1,025	0.40	1.46

**FIGURE 4:**
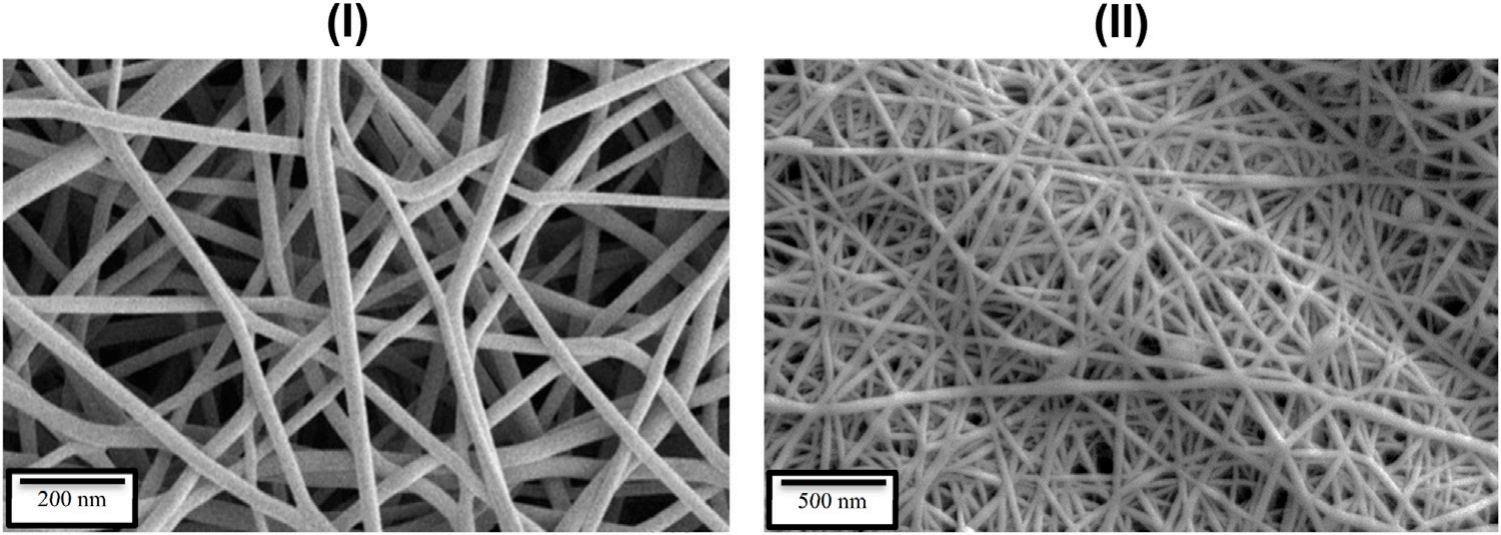
SEM image of polyvinylpyrrolidone nanofibers (I) and polyvinylpyrrolidone nanofibers containing methanol extract of *Daphne mucronata* (II).

Choosing the synthesis conditions of nanoparticles is very effective in their morphology and size. The SEM image of the synthesized polyvinylpyrrolidone nanofibers containing methanol extract of *D. mucronata* shows the same morphology of the composition (Figure 4). In addition, it can be concluded from the image that the synthesized polyvinylpyrrolidone nanofibers containing methanol extract of *D. mucronata* are in the range of nanostructures. Therefore, suitable conditions have been used in electrospinning to synthesize the desired product.

**FIGURE 5:**
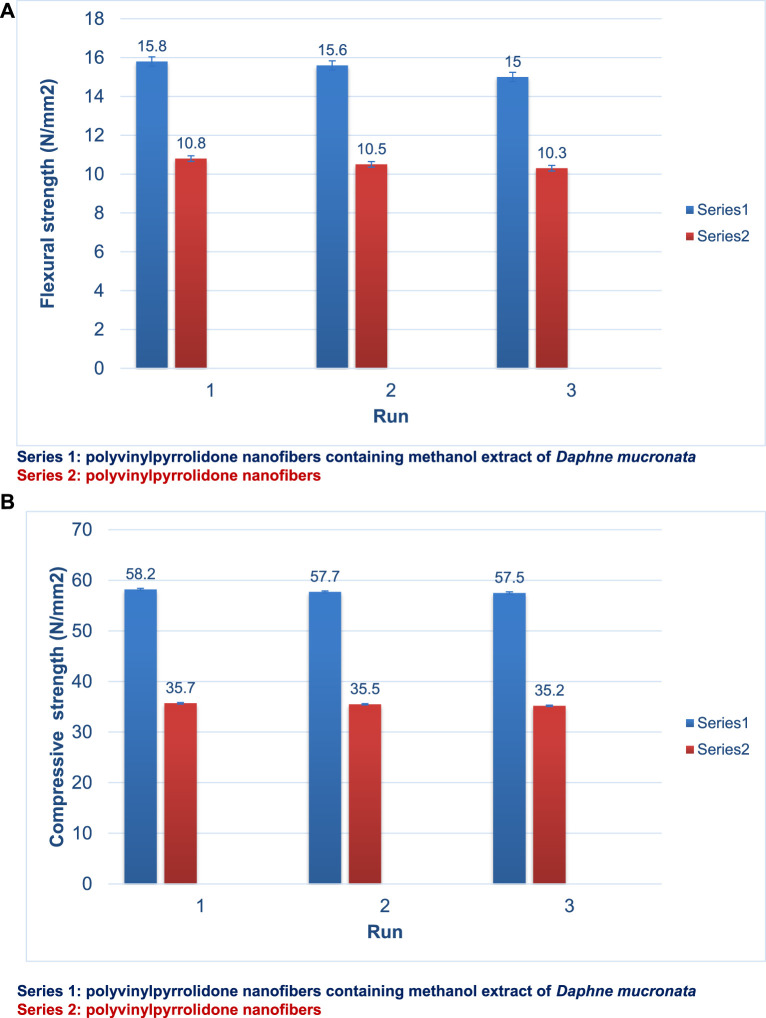
Flexural strength **(A)** and compressive strength **(B)** of polyvinylpyrrolidone nanofibers (Series 2) and polyvinylpyrrolidone nanofibers containing methanol extract of *Daphne mucronata* (Series 1).

Flexural strength and compressive strength are two other vital parameters in the properties of polymers and nanofibers. Flexural strength and compressive strength also depend on the synthesis method of the desired compound. Figure 5A displays the results of the flexural strength test for polyvinylpyrrolidone nanofibers (I) and polyvinylpyrrolidone nanofibers containing methanol extract of *D. mucronata* (II). The results show that the flexural strength of polyvinylpyrrolidone nanofibers containing methanol extract of *D. mucronata* (15.8 N/mm^2^) is higher than that of polyvinylpyrrolidone nanofibers (10.8 N/mm^2^). A lliterature review showed that the synthesized compound has a higher flexural strength than some of the previously synthesized similar compounds (Ngadiman et al., 2015; Su et al., 2022).

Figure 5B shows the compressive strength of polyvinylpyrrolidone nanofibers (I) and polyvinylpyrrolidone nanofibers containing methanol extract of *D. mucronata* (II). Here, too, polyvinylpyrrolidone nanofibers containing methanol extract of *D. mucronata* with 58.2 65 N/mm^2^ have a higher compressive strength than polyvinylpyrrolidone nanofibers with 35.7 65 N/mm^2^.

The high flexural strength and compressive strength of polyvinylpyrrolidone nanofibers containing methanol extract of *D. mucronata* compared to polyvinylpyrrolidone nanofibers can be attributed to the strong hydrogen bonds created in the final product based on the proposed structure.

The contact angle used for hydrophilicity showed 52^o^ for polyvinylpyrrolidone nanofibers and 38^o^ for polyvinylpyrrolidone nanofibers containing methanol extract of *D. mucronata*. A decrease in contact angle indicates an increase in hydrophilicity. Here too, according to the proposed structure for the final product, the decline in contact angle can be attributed to the many alcohol groups present in the final product, which have a high ability to perform hydrogen bonding with water molecules (Drelich et al., 2019; Hassan et al., 2019).

### 3.6 Antimicrobial evolution of polyvinylpyrrolidone nanofibers containing methanol extract of *Daphne mucronata*

As mentioned in the previous sections, the methanol extract of *D. mucronata* had high biological properties such as antimicrobial, antioxidant, and anticancer activity, and using it and polyvinylpyrrolidone, polyvinylpyrrolidone nanofibers containing methanol extract of *D. mucronata* were synthesized.

After confirming the structure of polyvinylpyrrolidone nanofibers containing methanol extract of *D. mucronata*, the synthesized nanofiber and polyvinylpyrrolidone nanofibers were subjected to antimicrobial tests on the previously studied strains. The results of the tests are given in Table 6.

**TABLE 6:** Antimicrobial activity of Polyvinylpyrrolidone nanofibers and Polyvinylpyrrolidone nanofibers containing methanol extract of *Daphne mucronata*.

Extracts species			Polyvinylpyrrolidone nanofibers	Polyvinylpyrrolidone nanofibers containing methanol extract of *Daphne mucronata*	Drug 1	Drug 1
Gram-positive	ATCC 11778	IZD	—	17.84	—	19.62
		MIC	—	32	—	4
		MBC	—	64	—	8
	ATCC 25729	IZD	—	—	—	14.97
		MIC	—	—	—	8
		MBC	—	—	—	16
	ATCC 29213	IZD	10.37	19.43	18.55	22.13
		MIC	2,048	16	16	4
		MBC	4,096	32	32	8
Gram negative	ATCC 13883	IZD	14.37	18.33	18.97	18.46
		MIC	128	4	2	8
		MBC	256	8	4	16
	ATCC 9610	IZD	10.83	20.99	19.42	20.61
		MIC	1,024	32	16	8
		MBC	2,048	32	32	16
	ATCC 19606	IZD	—	—	—	13.44
		MIC	—	—	—	64
		MBC	—	—	—	128
Aquatic and human	ATCC 29473	IZD	—	12.31	—	14.79
		MIC	—	128	—	32
		MBC	—	256	—	64
	ATCC 43921	IZD	11.73	18.29	17.81	17.17
		MIC	128	32	4	16
		MBC	512	64	8	32
	ATCC 29178	IZD	12.15	16.43	16.09	15.39
		MIC	256	32	8	32
		MBC	512	64	32	64
Fungal	ATCC 7601	IZD	9.94	18.66	—	18.43
		MIC	2,048	32	—	32
		MFC	4,096	64	—	64
	ATCC 10231	IZD	—	14.05	—	16.99
		MIC	—	128	—	64
		MFC	—	256	—	64
	ATCC 1022	IZD	10.16	15.29	—	17.34
		MIC	1,024	64	—	32
		MFC	2,048	128	—	64

MIC, MBC, and MFC, μg/mL; IZD, mm.

Drug 1, For bacteria, Cefazolin; For fungi, Tolnaftate.

Drug 2, For bacteria, Gentamicin; For fungi, Terbinafine.

The results showed that the presence of Gallic acid and Catechins in synthesized polyvinylpyrrolidone nanofibers containing methanol extract of *D. mucronata* increased its antimicrobial properties compared to polyvinylpyrrolidone nanofibers.

Here, Cefazolin, Gentamicin, Tolnaftate, and Terbinafine, which are well-known antibiotics in the market, were used to compare the antimicrobial activity of synthesized polyvinylpyrrolidone nanofibers containing methanol extract of *D. mucronata*.

The results proved that the synthesized nanofiber is more effective than commercial drugs on some strains. It is possible to mention bacterial and fungal strains such as *B. cereus* (ATCC 11778), *Y. ruckeri* (ATCC 29473), *F. oxysporum* (ATCC 7601), *C. albicans* (ATCC 10231), and *A. fumigatus Fresenius* (ATCC 1022), which synthesized nanofiber are effective on them, but Cefazolin and Tolnaftate as commercial drugs were not effective.

As can be seen from the results of Table 6, the effectiveness of polyvinylpyrrolidone nanofibers containing methanol extract of *D. mucronata* on the studied strains was more than the methanol extract of *D. mucronata*. The comparison of the antimicrobial activity of extract and nanofiber on the studied strains is given in Table 7.

**TABLE 7:** Comparison of antimicrobial activity of methanol extract of *Daphne mucronata* and Polyvinylpyrrolidone nanofibers containing methanol extract of *Daphne mucronata*.

Extracts species			Methanol extract of *Daphne mucronata*	Polyvinylpyrrolidone nanofibers containing methanol extract of *Daphne mucronata*
Gram-positive	ATCC 11778	IZD	15.27	17.84
		MIC	64	32
		MBC	128	64
	ATCC 25729	IZD	—	—
		MIC	—	—
		MBC	—	—
	ATCC 29213	IZD	16.49	19.43
		MIC	128	16
		MBC	256	32
Gram negative	ATCC 13883	IZD	12.04	18.33
		MIC	64	4
		MBC	128	8
	ATCC 9610	IZD	19.05	20.99
		MIC	128	32
		MBC	256	32
	ATCC 19606	IZD	—	—
		MIC	—	—
		MBC	—	—
Aquatic and human	ATCC 29473	IZD	9.79	12.31
		MIC	256	128
		MBC	512	256
	ATCC 43921	IZD	14.34	18.29
		MIC	256	32
		MBC	512	64
	ATCC 29178	IZD	14.91	16.43
		MIC	128	32
		MBC	256	64
Fungal	ATCC 7601	IZD	15.73	18.66
		MIC	64	32
		MFC	128	64
	ATCC 10231	IZD	12.61	14.05
		MIC	512	128
		MFC	1,024	256
	ATCC 1022	IZD	14.32	15.29
		MIC	256	64
		MFC	512	128

MIC, MBC, and MFC, μg/mL; IZD, mm.

As the final result of the comparison of the antimicrobial activity of the methanol extract of *D. mucronata* and nanofibers containing methanol extract of *D. mucronata*, it can be stated that the nanostructure with the high specific surface area has increased the antimicrobial activity of the nanofiber. The high specific surface area of fibrosis results in an increase in the contact surface of molecules with the studied strains and a rise in antimicrobial properties (Lam et al., 2021; Mammari et al., 2022).

## 4 Conclusion

The Iraqi Kurdistan Region *D. mucronata* was used in this study to prepare extracts of aqueous, ethanol, methanol, and hexane. Another goal of this study was the synthesis of nanofibers containing Iraqi Kurdistan Region *D. mucronata* with high biological properties. The extracts were investigated for their antibacterial, antifungal, antioxidant, and anticancer properties and compared for this purpose. The evaluations and comparisons showed that the highest effectiveness is related to the methanolic extract. So the results for methanolic extract in antimicrobial activity, MIC between 64 and 1,024 μg/mL, in antioxidant activity, IC_50_ 4.67 μg/mL, and in anticancer activity, IC_50_ 7.83 μg/mL were observed. Therefore, by using *D. mucronata* methanolic extract and polyvinylpyrrolidone with the electrospinning method, the desired nanofibers were synthesized. Characterizing and confirming the structure of the synthesized nanofibers was done using FTIR, TGA, BET, SEM, compressive strength, flexural strength, and hydrophilicity. Higher thermal stability (215.92°C), higher specific surface area (1,025 m^2^/g), higher flexural strength (15.8 N/mm^2^) and compressive strength (58.2 65 N/mm^2^) and higher hydrophilicity than polyvinylpyrrolidone nanofibers were among the characteristics of synthesized polyvinylpyrrolidone nanofibers containing methanol extract of *D. mucronata*. Which can be attributed to the creation of hydrogen bonds between the extract compounds and polyvinylpyrrolidone. Finally, the antimicrobial properties of nanofibers were also tested, and the results were compared with the antimicrobial properties of the extract. The comparison of the results showed that the nanofibers had higher and more significant properties than the extract due to the unique properties mentioned above, such as high specific surface area. Therefore, nanofiber containing the Iraqi Kurdistan Region *D. mucronata* and polyvinylpyrrolidone can be introduced as a nanofiber with potential biological properties.

## Data Availability

The original contributions presented in the study are included in the article/Supplementary Material, further inquiries can be directed to the corresponding author.

## References

[B1] Ahani Z. Nikbin M. Maghsoodlou M.-T. Farhadi-Ghalati F. Valizadeh J. Beyzaei H. (2018). Semi-synthesis, antibacterial and antifungal activities of three novel thiazolidin-4-one by essential oil of Anethum graveolens seeds as starting material. J. Iran. Chem. Soc. 15, 2423–2430. 10.1007/s13738-018-1431-y

[B2] Akhavan-Sigari R. Zeraati M. Moghaddam-Manesh M. Kazemzadeh P. Hosseinzadegan S. Chauhan N. P. S. (2022). Porous Cu-MOF nanostructures with anticancer properties prepared by a controllable ultrasound-assisted reverse micelle synthesis of Cu-MOF. BMC Chem. 16, 10. 10.1186/s13065-022-00804-2 35248138 PMC8898484

[B3] Alavi Rafiee S. Farhoosh R. Sharif A. (2018). Antioxidant activity of gallic acid as affected by an extra carboxyl group than pyrogallol in various oxidative environments. Eur. J. Lipid Sci. Technol. 120, 1800319. 10.1002/ejlt.201800319

[B4] Anwer M. K. Fatima F. Ahmed M. M. Aldawsari M. F. Alali A. S. Kalam M. A. (2022). Abemaciclib-loaded ethylcellulose based nanosponges for sustained cytotoxicity against MCF-7 and MDA-MB-231 human breast cancer cells lines. Saudi Pharm. J. 30, 726–734. 10.1016/j.jsps.2022.03.019 35812154 PMC9257851

[B5] Baig N. Kammakakam I. Falath W. (2021). Nanomaterials: a review of synthesis methods, properties, recent progress, and challenges. Mater. Adv. 2, 1821–1871. 10.1039/d0ma00807a

[B6] Beyzaei H. Deljoo M. K. Aryan R. Ghasemi B. Zahedi M. M. Moghaddam-Manesh M. (2018). Green multicomponent synthesis, antimicrobial and antioxidant evaluation of novel 5-amino-isoxazole-4-carbonitriles. Chem. Central J. 12, 1–8. 10.1186/s13065-018-0488-0 PMC6768021 30443685

[B7] Binkowska I. (2020). Hesperidin: synthesis and characterization of bioflavonoid complex. SN Appl. Sci. 2, 445. 10.1007/s42452-020-2256-8

[B8] Dias M. C. Pinto D. C. Silva A. M. (2021). Plant flavonoids: chemical characteristics and biological activity. Molecules 26, 5377. 10.3390/molecules26175377 34500810 PMC8434187

[B9] Dirar A. Alsaadi D. Wada M. Mohamed M. Watanabe T. Devkota H. (2019). Effects of extraction solvents on total phenolic and flavonoid contents and biological activities of extracts from Sudanese medicinal plants. South Afr. J. Bot. 120, 261–267. 10.1016/j.sajb.2018.07.003

[B10] Dodero A. Alberti S. Gaggero G. Ferretti M. Botter R. Vicini S. (2021). An up‐to‐date review on alginate nanoparticles and nanofibers for biomedical and pharmaceutical applications. Adv. Mater. Interfaces 8, 2100809. 10.1002/admi.202100809

[B11] Drelich J. W. Boinovich L. Chibowski E. Della Volpe C. Hołysz L. Marmur A. (2019). Contact angles: history of over 200 years of open questions. Surf. Innov. 8, 3–27. 10.1680/jsuin.19.00007

[B12] Edikresnha D. Suciati T. Munir M. M. Khairurrijal K. (2019). Polyvinylpyrrolidone/cellulose acetate electrospun composite nanofibres loaded by glycerine and garlic extract with *in vitro* antibacterial activity and release behaviour test. RSC Adv. 9, 26351–26363. 10.1039/c9ra04072b 35531031 PMC9070455

[B13] Etemadi Y. Shiri A. Eshghi H. Akbarzadeh M. Saadat K. Mozafari S. (2016). Synthesis, characterisation, and *in vitro* antibacterial evaluation of a new class of 2-substituted-4-methyl-7, 8-dihydro-5H-pyrimido [4, 5-d] thiazolo [3, 2-a] pyrimidines. J. Chem. Res. 40, 600–603. 10.3184/174751916x14737838285904

[B14] Fazal N. Khawaja H. Naseer N. Khan A. J. Latief N. (2020). Daphne mucronata enhances cell proliferation and protects human adipose stem cells against monosodium iodoacetate induced oxidative stress *in vitro* . Adipocyte 9, 495–508. 10.1080/21623945.2020.1812242 32867575 PMC7714443

[B15] Ghanadian M. Ali Z. Khan I. A. Balachandran P. Nikahd M. Aghaei M. (2020). A new sesquiterpenoid from the shoots of Iranian Daphne mucronata Royle with selective inhibition of STAT3 and Smad3/4 cancer-related signaling pathways. DARU J. Pharm. Sci. 28, 253–262. 10.1007/s40199-020-00336-x PMC7214574 32248516

[B16] Ghasemian Lemraski E. Jahangirian H. Dashti M. Khajehali E. Sharafinia S. Rafiee-Moghaddam R. (2021). Antimicrobial double-layer wound dressing based on chitosan/polyvinyl alcohol/copper: *in vitro* and *in vivo* assessment. Int. J. nanomedicine 16, 223–235. 10.2147/ijn.s266692 33469282 PMC7810733

[B17] Górniak I. Bartoszewski R. Króliczewski J. (2019). Comprehensive review of antimicrobial activities of plant flavonoids. Phytochem. Rev. 18, 241–272. 10.1007/s11101-018-9591-z

[B18] Han S. Li L.-Z. Song S.-J. (2020). Daphne giraldii Nitsche (Thymelaeaceae): phytochemistry, pharmacology and medicinal uses. Phytochemistry 171, 112231. 10.1016/j.phytochem.2019.112231 31901473

[B19] Hassan E. A. Yang L. Elagib T. H. Ge D. Lv X. Zhou J. (2019). Synergistic effect of hydrogen bonding and π-π stacking in interface of CF/PEEK composites. Compos. Part B Eng. 171, 70–77. 10.1016/j.compositesb.2019.04.015

[B20] Hosseinzadegan S. Hazeri N. Maghsoodlou M. T. Moghaddam-Manesh M. Shirzaei M. (2020). Synthesis and evaluation of biological activity of novel chromeno [4, 3-b] quinolin-6-one derivatives by SO 3 H-tryptamine supported on Fe 3 O 4@ SiO 2@ CPS as recyclable and bioactive magnetic nanocatalyst. J. Iran. Chem. Soc. 17, 3271–3284. 10.1007/s13738-020-01990-3

[B21] Kişla D. Gökmen G. G. Evrendilek G. Akan T. Vlčko T. Kulawik P. (2023). Recent developments in antimicrobial surface coatings: various deposition techniques with nanosized particles, their application and environmental concerns. Trends Food Sci. Technol. 135, 144–172. 10.1016/j.tifs.2023.03.019

[B22] Kurakula M. Rao G. K. (2020). Moving polyvinyl pyrrolidone electrospun nanofibers and bioprinted scaffolds toward multidisciplinary biomedical applications. Eur. Polym. J. 136, 109919. 10.1016/j.eurpolymj.2020.109919

[B23] Lam M. Migonney V. Falentin-Daudre C. (2021). Review of silicone surface modification techniques and coatings for antibacterial/antimicrobial applications to improve breast implant surfaces. Acta Biomater. 121, 68–88. 10.1016/j.actbio.2020.11.020 33212233

[B24] Lee B. K. Hyun S.-W. Jung Y.-S. (2020). Yuzu and hesperidin ameliorate blood-brain barrier disruption during hypoxia via antioxidant activity. Antioxidants 9, 843. 10.3390/antiox9090843 32916895 PMC7555663

[B25] Li Y. Zhu G. Xu X. Chen L. Lu T. Hill J. P. (2022). Embedding metal–organic frameworks for the design of flexible hybrid supercapacitors by electrospinning: synthesis of highly graphitized carbon nanofibers containing metal oxide nanoparticles. Small Struct. 3, 2200015. 10.1002/sstr.202200015

[B26] Lutfullah G. Shah A. Ahmad K. Haider J. (2019). Phytochemical screening, antioxidant and antibacterial properties of daphne mucronata. J. Traditional Chin. Med. 39, 764–771. 10.19852/j.cnki.jtcm.2019.06.002 32186146

[B27] Ma Y. Ding S. Fei Y. Liu G. Jang H. Fang J. (2019). Antimicrobial activity of anthocyanins and catechins against foodborne pathogens *Escherichia coli* and Salmonella. Food control. 106, 106712. 10.1016/j.foodcont.2019.106712

[B28] Mammari N. Lamouroux E. Boudier A. Duval R. E. (2022). Current knowledge on the oxidative-stress-mediated antimicrobial properties of metal-based nanoparticles. Microorganisms 10, 437. 10.3390/microorganisms10020437 35208891 PMC8877623

[B29] Manandhar S. Luitel S. Dahal R. K. (2019). *In vitro* antimicrobial activity of some medicinal plants against human pathogenic bacteria. J. Trop. Med. 2019, 1–5. 10.1155/2019/1895340 PMC6466868 31065287

[B30] Mehrali F. Ziyadi H. Hekmati M. Faridi-Majidi R. Qomi M. (2020). Kefiran/poly (vinyl alcohol)/poly (vinyl pyrrolidone) composite nanofibers: fabrication, characterization and consideration of effective parameters in electrospinning. SN Appl. Sci. 2, 895. 10.1007/s42452-020-2714-3

[B31] Moghaddam‐Manesh M. Beyzaei H. Heidari Majd M. Hosseinzadegan S. Ghazvini K. (2021). Investigation and comparison of biological effects of regioselectively synthesized thiazole derivatives. J. Heterocycl. Chem. 58, 1525–1530. 10.1002/jhet.4278

[B32] Moghaddam-Manesh M. Sargazi G. Roohani M. Zanjani N. G. Khaleghi M. Hosseinzadegan S. (2022). Synthesis of PVA/Fe3O4@ SiO2@ CPS@ SID@ Ni as novel magnetic fibrous composite polymer nanostructures and evaluation of anti-cancer and antimicrobial activity. Polym. Bull. 80, 11919–11930. 10.1007/s00289-022-04584-6

[B33] Mostafa A. A. Al-Askar A. A. Almaary K. S. Dawoud T. M. Sholkamy E. N. Bakri M. M. (2018). Antimicrobial activity of some plant extracts against bacterial strains causing food poisoning diseases. Saudi J. Biol. Sci. 25, 361–366. 10.1016/j.sjbs.2017.02.004 29472791 PMC5815983

[B34] Naseem T. Durrani T. (2021). The role of some important metal oxide nanoparticles for wastewater and antibacterial applications: a review. Environ. Chem. Ecotoxicol. 3, 59–75. 10.1016/j.enceco.2020.12.001

[B35] Nazir N. Muhammad J. Ghaffar R. Nisar M. Zahoor M. Uddin F. (2021). Phytochemical profiling and antioxidant potential of Daphne mucronata Royle and action against paracetamol-induced hepatotoxicity and nephrotoxicity in rabbits. Saudi J. Biol. Sci. 28, 5290–5301. 10.1016/j.sjbs.2021.05.051 34466107 PMC8381059

[B36] Ngadiman N. H. A. Noordin M. Idris A. Shakir A. S. A. Kurniawan D. (2015). Influence of polyvinyl alcohol molecular weight on the electrospun nanofiber mechanical properties. Procedia Manuf. 2, 568–572. 10.1016/j.promfg.2015.07.098

[B37] Nguyen N. H. Ta Q. T. H. Pham Q. T. Luong T. N. H. Phung V. T. Duong T.-H. (2020). Anticancer activity of novel plant extracts and compounds from Adenosma bracteosum (Bonati) in human lung and liver cancer cells. Molecules 25, 2912. 10.3390/molecules25122912 32599892 PMC7356985

[B38] Osanloo M. Arish J. Sereshti H. (2020). Developed methods for the preparation of electrospun nanofibers containing plant-derived oil or essential oil: a systematic review. Polym. Bull. 77, 6085–6104. 10.1007/s00289-019-03042-0

[B39] Panda L. Duarte-Sierra A. (2022). Recent advancements in enhancing antimicrobial activity of plant-derived polyphenols by biochemical means. Horticulturae 8, 401. 10.3390/horticulturae8050401

[B40] Park J.-A. Lee S.-C. Kim S.-B. (2019). Synthesis of dual-functionalized poly (vinyl alcohol)/poly (acrylic acid) electrospun nanofibers with enzyme and copper ion for enhancing anti-biofouling activities. J. Mater. Sci. 54, 9969–9982. 10.1007/s10853-019-03578-6

[B41] Popiel S. Nawała J. Dziedzic D. SoDerstroM M. Vanninen P. (2014). Determination of mustard gas hydrolysis products thiodiglycol and thiodiglycol sulfoxide by gas chromatography-tandem mass spectrometry after trifluoroacetylation. Anal. Chem. 86, 5865–5872. 10.1021/ac500656g 24831983

[B42] Rajamanickam K. Yang J. Sakharkar M. K. (2019). Gallic acid potentiates the antimicrobial activity of tulathromycin against two key bovine respiratory disease (BRD) causing-pathogens. Front. Pharmacol. 9, 1486. 10.3389/fphar.2018.01486 30662404 PMC6328469

[B43] Rhazi L. Depeint F. Ayerdi Gotor A. (2022). Loss in the intrinsic quality and the antioxidant activity of sunflower (Helianthus annuus L.) oil during an industrial refining process. Molecules 27, 916. 10.3390/molecules27030916 35164180 PMC8839766

[B44] Shah A. Lutfullah G. Ahmad K. Khalil A. T. Maaza M. (2018). Daphne mucronata-mediated phytosynthesis of silver nanoparticles and their novel biological applications, compatibility and toxicity studies. Green Chem. Lett. Rev. 11, 318–333. 10.1080/17518253.2018.1502365

[B45] Shahryari T. Alizadeh V. Kazemzadeh P. Jadoun S. Chauhan N. P. S. Sargazi G. (2022). A controllable procedure for removing Navicula algae from drinking water using an ultrasonic-assisted electrospun method for highly efficient synthesis of Co-MOF/PVA polymeric network. Appl. Phys. A 128, 396. 10.1007/s00339-022-05524-x

[B46] Su S. Hu S. Liu Q. (2022). Application of polypyrrole cellulose nanocrystalline composite conductive material in garment design. Adv. Mater. Sci. Eng. 2022, 1–11. 10.1155/2022/4187826

[B47] Sun Q. Yao G.-D. Song X.-Y. Qi X.-L. Xi Y.-F. Li L.-Z. (2017). Autophagy antagonizes apoptosis induced by flavan enantiomers from Daphne giraldii in hepatic carcinoma cells *in vitro* . Eur. J. Med. Chem. 133, 1–10. 10.1016/j.ejmech.2017.03.072 28371676

[B48] Tanase C. Coșarcă S. Muntean D.-L. (2019). A critical review of phenolic compounds extracted from the bark of woody vascular plants and their potential biological activity. Molecules 24, 1182. 10.3390/molecules24061182 30917556 PMC6470986

[B49] Wu S. Li K. Shi W. Cai J. (2022). Preparation and performance evaluation of chitosan/polyvinylpyrrolidone/polyvinyl alcohol electrospun nanofiber membrane for heavy metal ions and organic pollutants removal. Int. J. Biol. Macromol. 210, 76–84. 10.1016/j.ijbiomac.2022.05.017 35533844

[B50] Wu S. Shi W. Li K. Cai J. Xu C. Gao L. (2023). Chitosan-based hollow nanofiber membranes with polyvinylpyrrolidone and polyvinyl alcohol for efficient removal and filtration of organic dyes and heavy metals. Int. J. Biol. Macromol. 239, 124264. 10.1016/j.ijbiomac.2023.124264 37003384

[B51] Yadav D. Amini F. Ehrmann A. (2020). Recent advances in carbon nanofibers and their applications–a review. Eur. Polym. J. 138, 109963. 10.1016/j.eurpolymj.2020.109963

[B52] Zahrani N. a.A. El-Shishtawy R. M. Asiri A. M. (2020). Recent developments of gallic acid derivatives and their hybrids in medicinal chemistry: a review. Eur. J. Med. Chem. 204, 112609. 10.1016/j.ejmech.2020.112609 32731188

