# Supplementary material for: Methanol extract of Iraqi Kurdistan Region Daphne mucronata as a potent source of antioxidant, antimicrobial, and anticancer agents for the synthesis of novel and bioactive polyvinylpyrrolidone nanofibers
Source: Front Chem. 2023 Oct 25;11:1287870. doi: 10.3389/fchem.2023.1287870 (PMC10634434; doi:10.3389/fchem.2023.1287870)
Supplement: Supplementary file 1 [file DataSheet1.PDF]

Supplementary Material

Methanol extract of Iraqi Kurdistan Region *Daphne mucronata* as potent source of antioxidant, antimicrobial, and anticancer agents for the synthesis of novel and bioactive Polyvinylpyrrolidone nanofibers

Khursheed Muzammil, Mazin Hadi Kzar, Faraj Mohammed, Zahraa Ibrahim Mohammed, Sarah A. Hamood, Talib Kh. Hussein, Saheb Jubeir Hanoon, Maytham T. Qasim, Ali Alsalamy*

*** Correspondence:** Ali Alsalamy, alsalamyali06@gmail.com

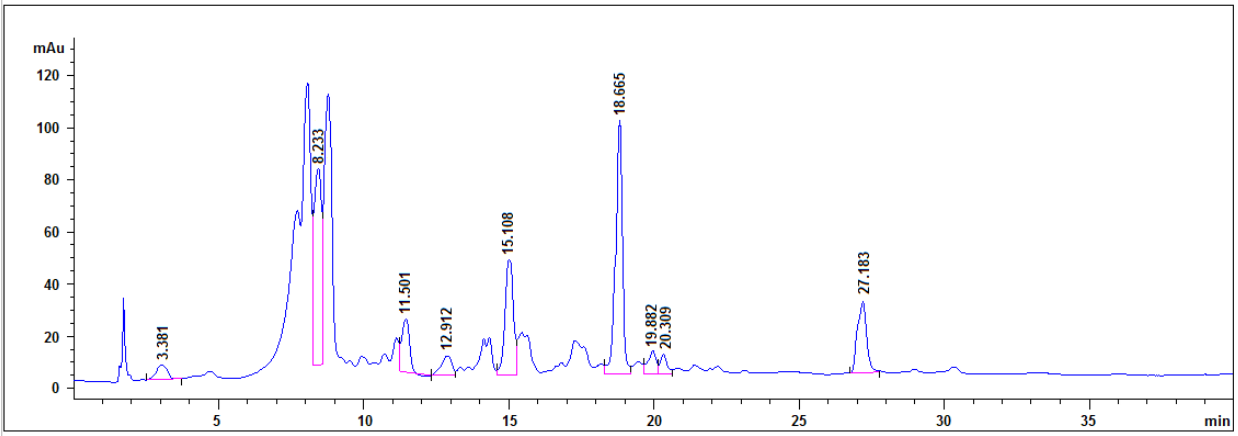

Supplementary Figure 1. Gas chromatogram of methanol *Daphne mucronata* extract

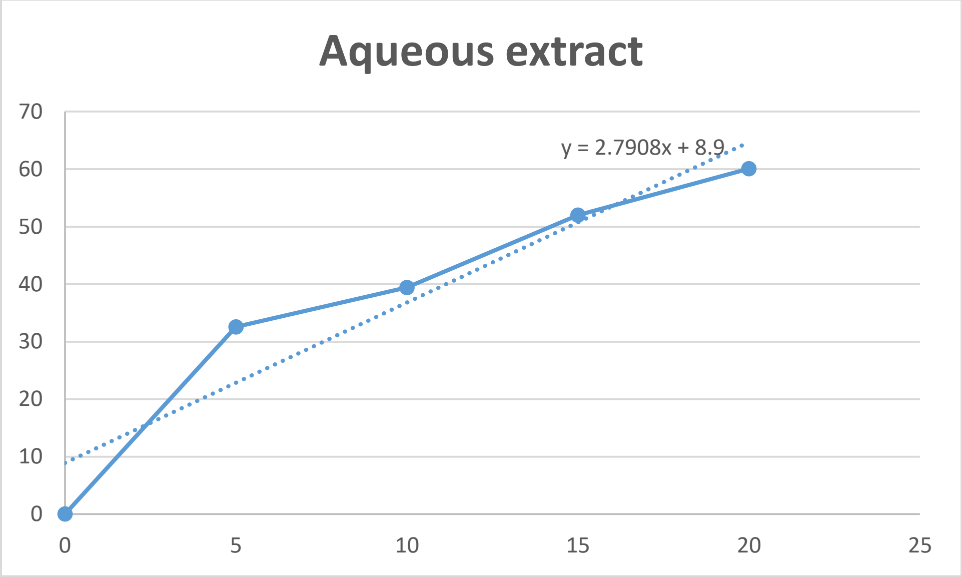
Aqueous extract
70
60
50
40
30
20
10
0
0
5
10
15
20
25
y = 2.7908x + 8.9

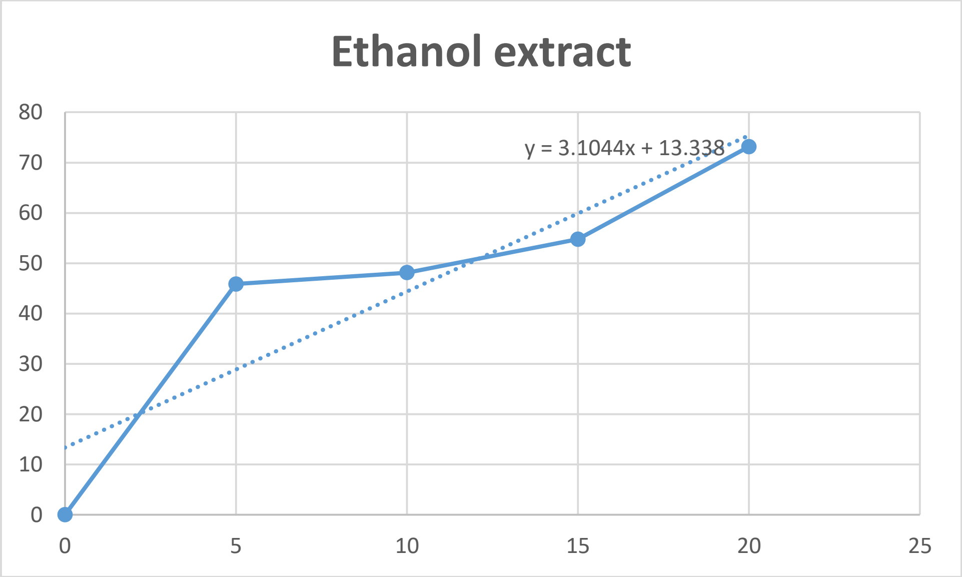
Ethanol extract
80
70
60
50
40
30
20
10
0
0
5
10
15
20
25
y = 3.1044x + 13.338

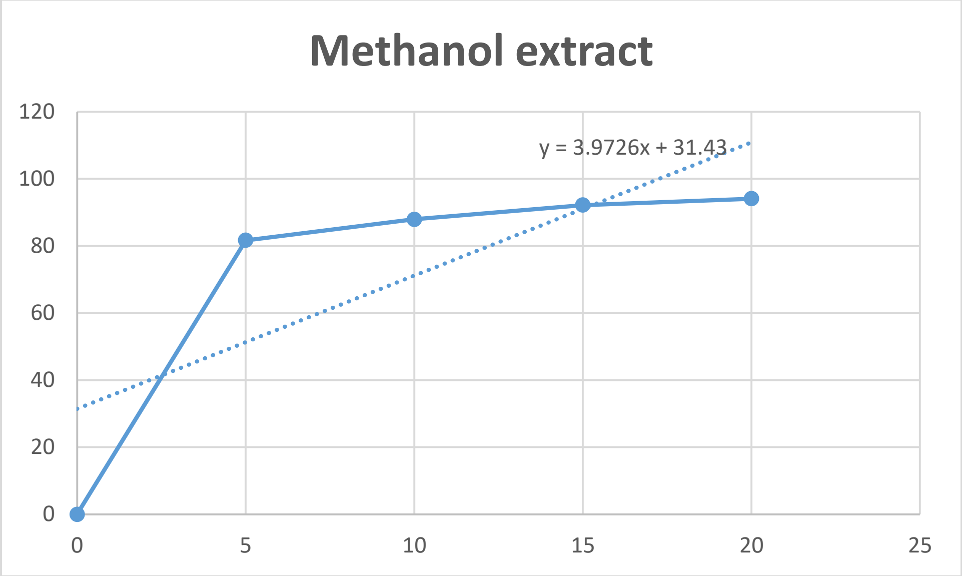
Methanol extract
y = 3.9726x + 31.43
120
100
80
60
40
20
0
0
5
10
15
20
25

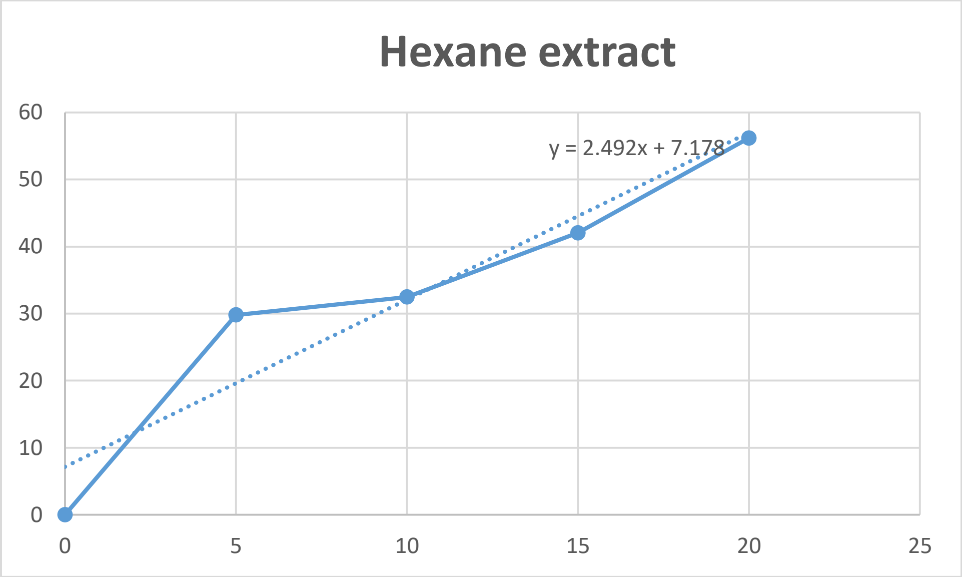
Hexane extract
y = 2.492x + 7.178
60
50
40
30
20
10
0
0
5
10
15
20
25

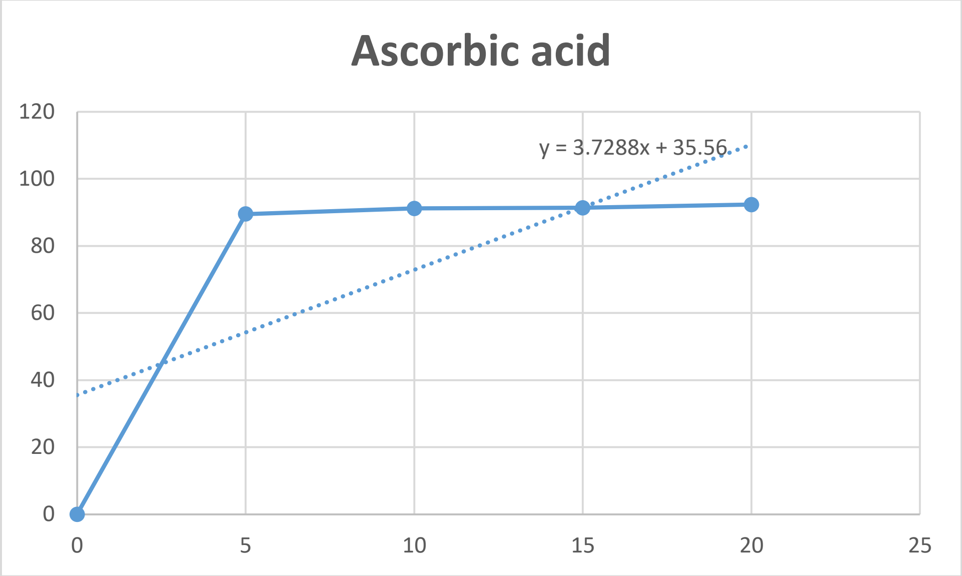

Supplementary Figure 2. Percentage inhibition/concentration curves of aqueous, ethanol, methanol, and hexane *Daphne mucronata* extracts in antioxidant evolution
